# ﻿A new species of *Iochroma* Benth. (Solanaceae) from the eastern Andes of Colombia

**DOI:** 10.3897/phytokeys.232.108474

**Published:** 2023-09-18

**Authors:** Andrés Orejuela, Stacey D. Smith, Boris Villanueva, Rocío Deanna

**Affiliations:** 1 Grupo de Investigación en Recursos Naturales Amazónicos-GRAM, Facultad de Ingenierías y Ciencias Básicas and Herbario Etnobotánico del Piedemonte Andino Amazónico (HEAA), Instituto Tecnológico del Putumayo-ITP, Mocoa, Putumayo, Colombia Jardín Botánico de Bogotá José Celestino Mutis Bogotá Colombia; 2 Herbario JBB, Subdirección científica, Jardín Botánico de Bogotá José Celestino Mutis, Bogotá D.C., Colombia Instituto Tecnológico del Putumayo-ITP Mocoa Colombia; 3 Department of Ecology and Evolutionary Biology, University of Colorado, Boulder, Colorado, USA University of Colorado Boulder United States of America; 4 Instituto Multidisciplinario de Biología Vegetal (IMBIV), CONICET-UNC, Universidad Nacional de Córdoba, CC 495, 5000, Córdoba, Argentina Universidad Nacional de Córdoba Córdoba Argentina

**Keywords:** Andes, Colombia, *Iochroma*, Iochrominae, IUCN Red List, Solanaceae

## Abstract

*Iochromaorozcoae* A.Orejuela & S.D.Sm., **sp. nov.** (Solanaceae) is described from the Andean forests of Cundinamarca in the eastern cordillera of Colombia. *Iochromaorozcoae* was first collected by the eminent Spanish priest and botanist José Celestino Mutis in the late part of the 18^th^ century, but the specimens have lain unrecognised in herbaria for over 200 years. The species shares many features with its closest relative, *Iochromabaumii* S.D.Sm. & S.Leiva, but it differs from it in having small flowers with five corolla lobes and few inflorescences per branch, located near the shoot apex with 1 to 4 (–8) flowers, fruits that are greenish-yellow when ripe and its restricted geographic distribution. A description of *I.orozcoae* is provided, along with a detailed illustration, photographs of live plants, a comparison with closely-related species and a key to all Colombian species of *Iochroma* Benth. In closing, we emphasise the value of historical collections for the knowledge of biodiversity.

## Abstract (es)

*Iochromaorozcoae* A.Orejuela & S.D.Sm., **sp. nov.** (Solanaceae) se describe a partir de los bosques andinos de Cundinamarca, en la cordillera oriental de Colombia. *Iochromaorozcoae* fue recolectada por primera vez por el eminente sacerdote y botánico español José Celestino Mutis a finales del siglo XVIII, pero los especímenes han permanecido sin ser reconocidos en herbarios durante más de 200 años. La especie comparte muchas características con su pariente más cercano, *Iochromabaumii* S.D.Sm. & S.Leiva, pero difiere de esta en sus flores más pequeñas con cinco lóbulos de la corola y pocas inflorescencias por rama ubicadas cerca del ápice de la rama, con 1 a 4 (–8) flores por inflorescencia, frutos verde-amarillentos al madurar, y su distribución geográfica restringida. Se presenta una descripción de *I.orozcoae*, junto con una ilustración detallada, fotografías en vivo, una comparación con las especies cercanamente relacionadas y una clave para las especies colombianas de *Iochroma* Benth. Finalmente, enfatizamos el valor de las colecciones históricas para el conocimiento de la biodiversidad.

## ﻿Introduction

*Iochroma* Benth. (Solanaceae) is a neotropical genus that comprises approximately 30 species distributed from Colombia to Peru, with the highest concentration of species found in the Peruvian Andes (Smith and Baum 2006). Members of *Iochroma* are unarmed shrubs and small trees that display showy, tubular flowers and ovoid berries with an enlarging calyx. These species are typically found in cloud forest clearings and disturbed areas between 1800 and 2800 m altitude. Their flowers are pollinated by hummingbirds and insects (Smith and Baum 2006; Smith et al. 2008). The recently published Catalogue of the Plants and Lichens of Colombia (Bernal et al. 2016) reported three native species for Colombia, the red-flowered *Iochromafuchsioides* Miers and *Iochromagesnerioides* (Kunth) Miers and the white-flowered *Iochromaarborescens* (L.) J.M.H.Shaw. Additionally, a recently-described Ecuadorian species, *I.baumii* S.D.Sm. & S.Leiva, has been mentioned for Colombia. Initially documented from a single specimen collected by Cuatrecasas in Caldas in 1946 (Smith and Leiva 2011), its presence has been further confirmed by a subsequent collection in a nearby locality during 2022. In addition to these species, we have made the remarkable rediscovery of an unusual *Iochroma* species that was first collected by José Celestino Mutis over 200 years ago during the Royal Botanical Expedition of the New Kingdom of Granada (1783–1816). This species does not correspond to any currently-recognised species within the genus. Recent collection efforts in the Municipality of Lenguazaque, Cundinamarca, located in the eastern Andes of Colombia and herbarium work have provided us with comprehensive material, enabling us to confirm the novelty and relationships of this species. Here, we provide a description of this species, *Iochromaorozcoae* A.Orejuela & S.D.Sm., sp. nov. along with a detailed comparison to its closest relatives, based on a phylogenetic analysis. We include an assessment of its conservation status and a dichotomous key for all *Iochroma* species distributed in Colombia to aid in identification.

## ﻿Material and method

﻿All specimens of the genus *Iochroma* from the Colombian herbaria COL, PSO, JBB and FMB and Ecuadorian herbaria QCA and QCNE (acronyms follow Index Herbariorum http://sweetgum.nybg.org/science/ih/) were revised to understand morphological variation across the genus, as well as major international herbaria that hold representatives from countries across the Andes (BM, E, F, K, MO). Herbarium material of the new species was collected in 2017 and 2021 and deposited at Herbario del Jardín Botánico de Bogotá (JBB) and Herbario Nacional Colombiano (COL). Flowers and fruits were preserved in 70% alcohol to facilitate preparation of taxonomic descriptions and illustrations.

For phylogenetic analysis, DNA was extracted from silica gel dried leaf material and three nuclear markers were sequenced (internal transcribed spacer region (ITS), the granule-bound starch synthase (GBSSI or waxy) gene and the second intron of *LEAFY* (*LFY*)), following Deanna et al. (2019). GenBank accession numbers for these sequences are MH763720, MH796580 and MH82214, respectively. We added these sequences to the dataset of Deanna et al. (2019) and carried out a partitioned Maximum Likelihood phylogenetic analysis in the RAxML blackbox (https://raxml-ng.vital-it.ch/). We chose GTR+gamma as the model of sequence evolution and assessed clade support with automatic bootstrapping (cut-off of 0.03).

To map the distribution of the new species and its close relatives, specimens with coordinates were mapped directly and those lacking coordinates were located using Google Earth, GeoNames gazetteer (http://www.geonames.org) and GEOLocate Web service (https://www.geo-locate.org/default.html). Distribution maps were created using QGIS (QGIS Development Team 2023). Conservation assessments were made, based on the IUCN Red List Categories and Criteria (IUCN 2012) and the most recent guidelines for using the IUCN Red List Categories and Criteria (IUCN 2022). Herbarium material, field observations and photos were all used to construct the identification key.

## ﻿Taxonomic treatment

### ﻿ Iochroma orozcoae

Taxon classificationPlantaeSolanalesSolanaceae

A.Orejuela & S.D.Sm. sp. nov.

751BAFA3-C212-5219-8AD0-EA94AC643259

urn:lsid:ipni.org:names:77326965-1

Figs 1 , 2

#### Type.

**Colombia. Cundinamarca**: vía Ubate-Lenguazaque, carretera sin pavimentar, antes del sector conocido como las balsas, 5°20'2.5"N, 73°43'23"W, 2600 m elev., 27 August 2017, *A. Orejuela & J. Castillo 2942* [holotype: JBB, (accession #JBB30649); isotypes: COL, HUA, HEAA].

**Figure 1.**
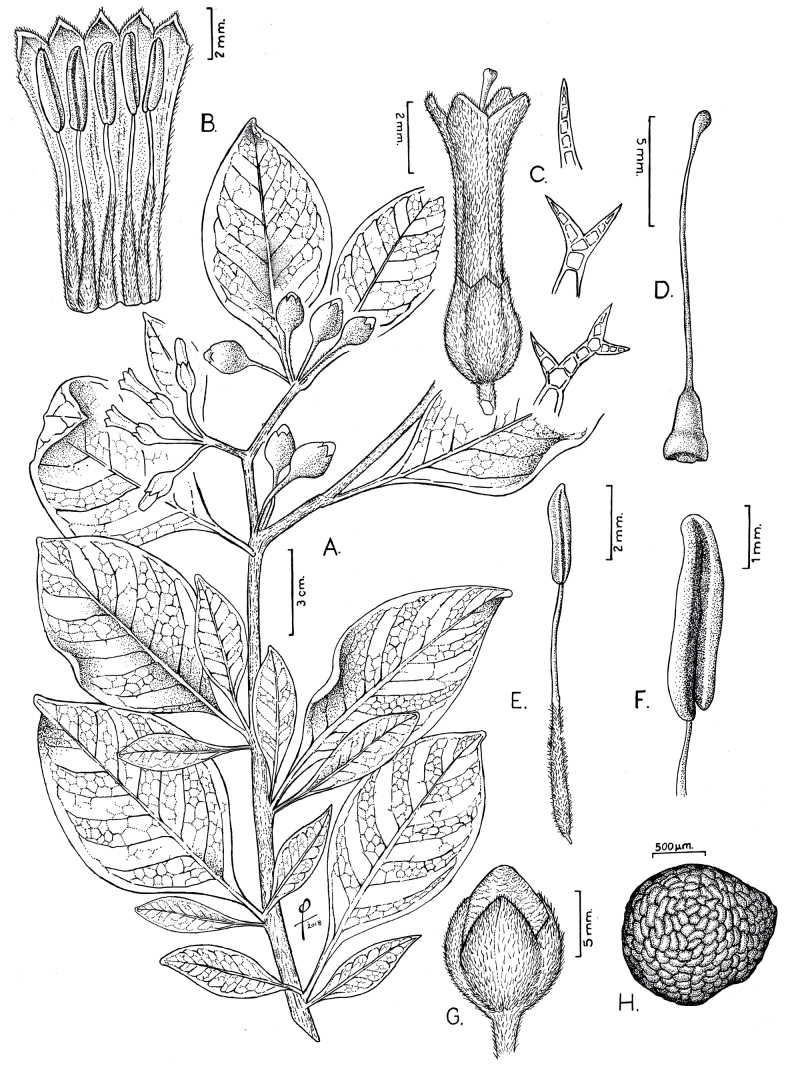
*Iochromaorozcoae* A.Orejuela & S.D.Sm. **A** flowering branch **B** inner corolla surface, showing the stamens **C** flower. Detail is shown for the simple and branched hairs on the corolla **D** gynoecium **E, F** details of the stamens and anthers **G** fruit with persistent calyx **H** seed. Drawn by Omar Bernal from Orejuela & Castillo 2942 & Orejuela et al. 3407.

#### Diagnosis.

A *Iochromabaumii* S.D.Sm. & S.Leiva affinis, sed paucarum inflorescentiae in ramum prope apicem germinis dispositae sunt, quae 1 ad 4 (–8) floribus, flores minores 1.5–2 cm longi; corolla quinque lobis constat, et fructus viridis-flavus colore maturo differt.

**Figure 2.**
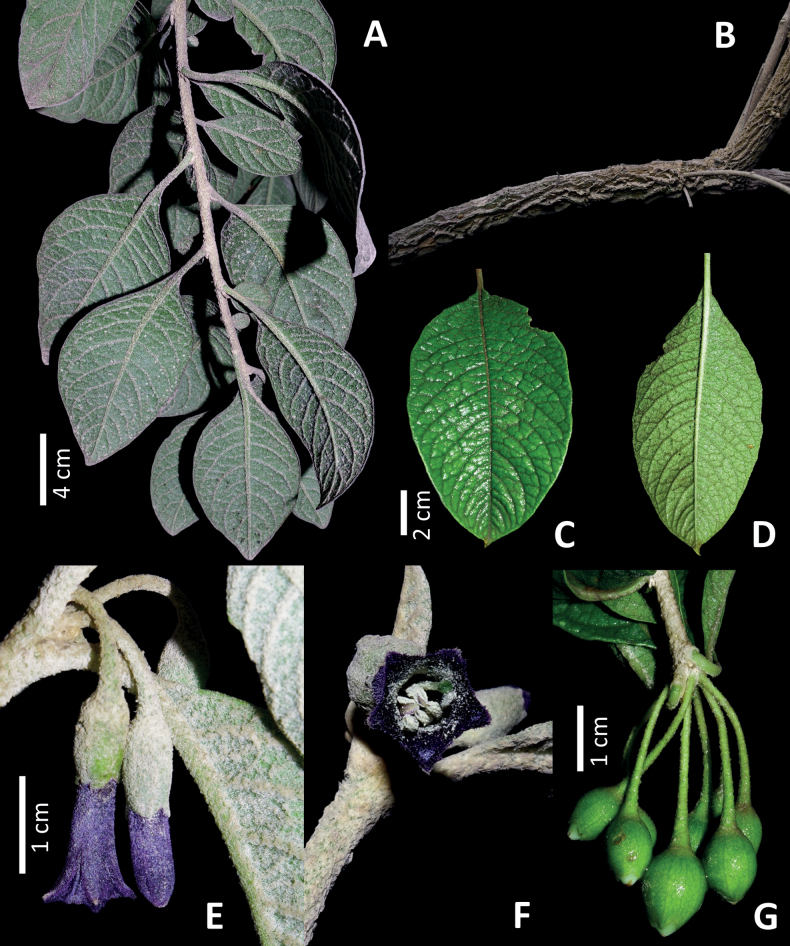
*Iochromaorozcoae* A.Orejuela & S.D.Sm. **A** young branch **B** stem detail **C, D** leaf detail showing the abaxial and adaxial sides **E** floriferous branch with flowers in lateral view **F** floriferous branch with a flower in frontal view **G** fruits showing the accrescent appressed calyx. Photos by Andrés Orejuela.

#### Description.

Shrub 1.5–3 m tall; young stems pubescent with branched hairs, older stems with corky bark. Leaves alternate, simple, (2.1–) 5.3–11.6 × (1.2–) 2.2–5.5 cm, elliptic; adaxial surface glabrescent; abaxial surface densely pubescent with branched hairs; base cuneate; margins entire; apex acute to attenuate; petioles (0.4–) 0.8–2.5 (–3) cm. Inflorescences axillary on young branches near the shoot apex, 1 to 4 (–8)-flowered; pedicels 1.1–1.7 (–2) cm in flower, 1.6–2.4 cm in fruit, terete, pendulous, densely pubescent with many-branched hairs. Calyx 6–7 × 5–6 mm, tubular to slightly urceolate, green, with few to many-branched hairs, with five broadly triangular lobes, ca. 0.8 × 2–2.5 mm in flower, shallowly divided in flower, deeply divided in fruit, in fruit, the calyx accrescent 9–10 × 10–11 mm, lobes 4–5 × 7–8 mm; corolla 15–20 × 4–4.5 mm at anthesis, tubular, flaring at the mouth, the exterior deep blue-purple, with many simple or occasionally branched hairs, the pubescence increasing towards the mouth, the interior deep blue-purple, glabrous, the lobes 5, 2.6–2.8 × 2.2–2.6 mm, acute to the apex and cucullate; stamens 5, included; filaments with simple and branched hairs, fused to the corolla at 3.8–5.2 mm from the base, with the free portion 7–11.4 mm long; anthers 3.5–5.2 × 1.3–1.7 mm, oblong, cream, longitudinally dehiscent; ovary 3.6–4.8 × 1.7–2.5 mm, pyriform, glabrous, with a yellow nectariferous disc, style 13–15 mm, the stigma green, clavate, bilobed. Berry 12–14 × 9–11 mm, slightly ovoid, greenish-yellow at maturity with 20 to 30 sclerosomes, the basal 3/4 enveloped in the accrescent fruiting calyx; seeds 110 to 170 per berry, 2–2.1 × 1.7–1.9 mm, yellow, reniform.

#### Etymology.

This species is named in honour of Clara Inés Orozco Pardo, an Associate Professor of botany at the Instituto de Ciencias Naturales of the Universidad Nacional de Colombia. Her dedication to the understanding of Colombian flora, particularly in the fields of taxonomy and systematics of the Brunelliaceae and Solanaceae families, has been remarkable. She has also played a crucial role in mentoring several Colombian botanists, including the first author of this paper.

#### Distribution and ecology.

**Figure 3.**
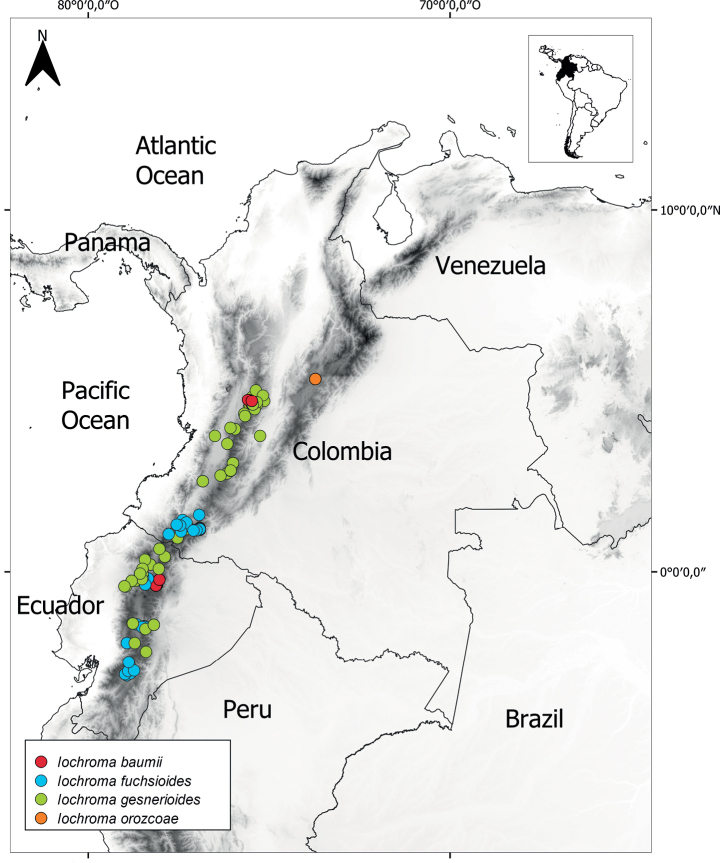
Geographic distribution of *I.orozcoae* (orange circles) and closely-related species in the “F” clade with presence in Colombia, including *I.baumii* (red circles), *I.gesnerioides* (green circles) and *I.fuchsioides* (blue circles).

*Iochromaorozcoae* is found in the Municipality of Lenguazaque, Cundinamarca Department, in the eastern Andes of Colombia, in the surroundings of the rock formation known as the Farallones de Lenguazaque, which is situated at an elevation of 2600 m (Fig. 3). The Farallones de Lenguazaque exhibit a vegetation type characteristic of high-altitude mountain ecosystems. The primary vegetation in this area comprises high Andean forest relicts of native forest, secondary forest, plantations of foreign species, grasslands and subparamo vegetation.

#### Phylogeny.

**Figure 4.**
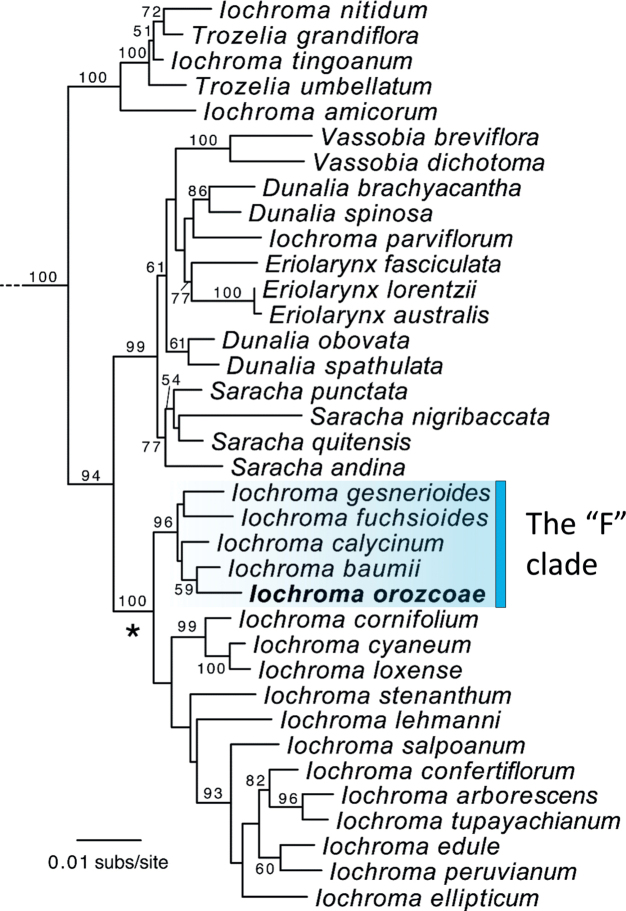
Maximum Likelihood phylogeny of Iochrominae including the new species, *I.orozcoae*. Relationships based on three nuclear markers with taxon sampling following Deanna et al. (2019). Physalideae outgroups from Smith and Baum (2006) (*Physalisperuviana* L., *Leucophysalisgrandiflora* (Hook.) Rydb., *Witheringiasolanacea* L’Hér., *Tubocapsicumanomalum* (Franch. & Sav.) Makino and *Depreasachapapa* (Hunz.) S.Leiva & Deanna) were used to root the tree and pruned for visual purposes. Bootstrap values above 50% are shown. Nomenclatural rearrangements are underway for Iochrominae (Deanna et al., unpublished) given the non-monophyly of the genera. The clade denoted with an asterisk will remain assigned to the genus *Iochroma* as it contains the type species (*Iochromacyaneum* (Lindl.) M.L. Green ex G.H.M. Lawr. & J.M. Tucker) and most of the species described in this genus.

*Iochromaorozcoae* belongs to the “F” clade (Smith and Baum 2006) containing other northern Andean species (*I.gesnerioides*, *I.fuchsioides*, *I.calycinum* Benth. and *I.baumii*) with high bootstrap support (96%). The red-flowered species (*I.gesnerioides* and *I.fuchsioides*) are separated from the blue-flowered species (*I.calycinum*, *I.baumii* and *I.orozcoae*), but that split is not well supported. Amongst the blue-flowered members of the “F” clade, *I.baumii* and *I.orozcoae* appear as sister taxa with 59% bootstrap support (Fig. 4).

#### Preliminary conservation status.

*Iochromaorozcoae* is classified as a data-deficient species (DD) due to the limited information on its abundance and distribution that is inadequate for comprehensively assessing its conservation status. The species has been collected only on four occasions, suggesting probable local rarity. The initial collection was made by José Celestino Mutis in 1783 from an unknown locality, followed by another by Oscar Haught in 1947 from Lenguazaque, Cundinamarca and a couple of recent collections by the first author from the same locality as Haught’s collection. Despite conducting an extensive search through specialised literature, the drawings of the Royal Botanical Expedition of the New Kingdom of Granada (1783–1816) and Mutis’ journals, the precise location of Mutis’ first collection remains unknown. We infer that the distribution of the species may be highly restricted, based on the small number of known collections from the same locality. Assumptions regarding its scarcity and restricted distribution, however, require further studies. Living plants of *I.orozcoae* originated from seeds, collected at the type locality and are conserved ex situ in the living collections of the Jardín Botánico de Bogotá.

#### Discussion.

**Figure 5.**
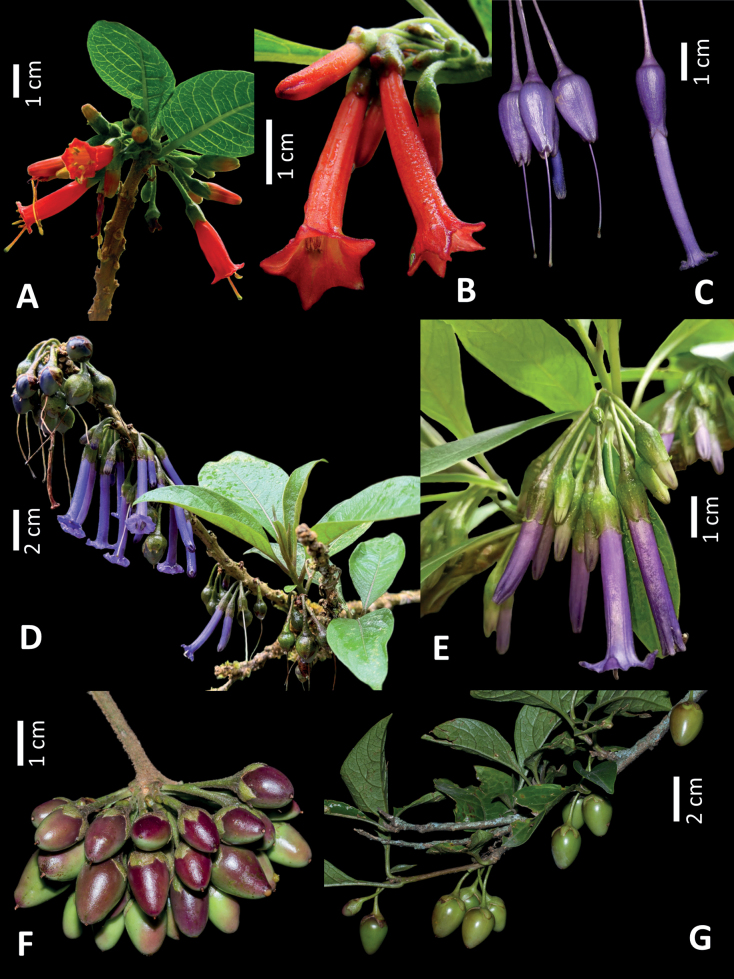
Species within the “F” clade of *Iochroma* that are closely related to *I.orozcoae***A** flowering branch of *I.fuchsioides* observed at Parque Recreacional y Bosque Protector Jerusalem, Malchingui, Ecuador **B** flowers of *I.gesnerioides* photographed at the Jardín Botánico de Bogotá, Colombia **C** flowers of *I.calycinum* at the reserva Otonga, Sigchos, Cotopaxi, Ecuador **D** flowers and fruits of *I.baumii* observed close to the type locality in Papallacta-Baeza road, Quijos, Napo, Ecuador **E** flowering branch of *I.baumii* from populations from La Pastora, Caldas, Colombia **F** fruits of *I.gesnerioides* photographed at the Jardín Botánico de Bogotá, Colombia **G** fruits of *I.fuchsioides* photographed close to Pasto, Nariño, Colombia. Photos by Andrés Orejuela (**B, C, F, G**), Hal Mitchell (**D**), Nathaly Obregón (**A**) and Juan David Tovar (**E**).

Our phylogenetic analyses offer strong support for the placement of *I.orozcoae* in the northern Andean “F” clade from Smith and Baum (2006). However, it differs from all other species in its broader and denser pubescence on both its vegetative and reproductive parts, small corollas and less abundant inflorescences with fewer flowers (Figs 1, 2, 5). The new species shares with *I.baumii* and *I.calycinum* intensely pigmented flowers, which are variously described as purple, violet or blue (Fig. 5). In contrast to *I.baumii* and *I.fuchsioides* which have corollas with ten teeth (five major lobes and five smaller teeth alternating with the lobes), *I.orozcoae* has corollas with five teeth corresponding to the lobes. *Iochromaorozcoae* is morphologically more similar to *I.baumii*. However, the new species is easily separated from *I.baumii* because it presents smaller flowers, 1.5–2 cm long (versus 2.8–4.5 cm long), only a few inflorescences per branch, located near the shoot apex with 1 to 4 (–8) flowers (versus masses of inflorescences per branch in clusters typically on older, often leafless branches, rarely near the shoot apex, with 6 to 12 flowers per inflorescence), corolla with five lobes (versus a 10-lobed corolla) and, while the fruits of *I.orozcoae* ripen in greenish-yellow colour, those of *I.baumii* are greenish-purple when ripe. A detailed comparison between *I.orozcoae* and the remaining species in the “F” clade can be found in Table 1.

**Table 1.** A morphological and geographical comparison of *Iochroma* species in the “F” clade (sensu Smith and Baum (2006)), including the new species *I.orozcoae*.

	*Iochromaorozcoae*	*Iochromabaumii*	*Iochromagesnerioides*	*Iochromafuchsioides*	*Iochromacalycinum*
**Geographical distribution**	Eastern Colombia	Ecuador and central Colombia	Ecuador and southern and central Colombia	Peru, Ecuador and southern Colombia	Ecuador and Peru
**Habitat**	Partially altered high Andean forests and roadsides to 2600 m	Disturbed cloud forest habitats, such as pasture hedges, forest gaps and roadsides from 2600 to 3300 m	Common in middle to high elevations in cloud forests, pastures hedges and roadsides from 1800 to 3300 m	Common in middle to high elevations in cloud forests, pastures hedges and roadsides from 2400 to 3500 m	Common in some areas of the wet cloud forest from 2000 to 3350 m
**Leaf size (cm)**	(2.1–) 5.3–11.6 × (1.2–) 2.2–5.5	(6–) 9–17 × (2.5–) 3–6	12–18 (–30) × 5–7.5 (–12)	3.5–7 (–9) × 1.5–3 (–5)	12–23 × 5–9
**Leaf shape**	Elliptic	Elliptic to lanceolate	Elliptic to lanceolate	Obovate to elliptic	Elliptic to lanceolate
**Inflorescence**	Inflorescences axillary on young branches near the shoot apex, 1 to 4 (–8)-flowered	Inflorescences axillary, typically on older, often leafless branches, rarely near the shoot apex, 6 to 12-flowered	Inflorescences axillary, in clusters with masses of flowers (with up ca. 120 flowers) along older or upper leaf nodes	Inflorescences axillary, in small clusters of flowers (1 to 12 flowers) on young branches near the shoot apex or upper leaf nodes	Inflorescences axillary, in small clusters of flowers on young branches near the shoot apex (5 to 12 flowers) or older branches (1 to 6 flowers)
**Calyx in flower**	Calyx 6–7 × 5–6 mm, slightly urceolate, green, with densely branched hairs	Calyx 4–9 × 3.8–6 mm, tubular to slightly urceolate, purplish-green, with few to many branched hairs	Calyx 3–5 × 4–5 mm, cup-shaped, green, with densely branched hairs	Calyx 7–13 × 4.5–5.5 mm, tubular to campanulate, green, hairless or with a few scattered branched hairs	Calyx 25 × 8–17 mm, inflated, elliptic, purplish, hairless or with a few branched hairs
**Corolla**	Corolla 15–20 × 4–4.5 mm, deep blue-purple, 5-lobed	Corolla 28–45 × 4–6 mm, deep blue-purple, 10-lobed	Corolla 25–40 × 5–6 mm, red, orange-red, orange to salmon, 5-lobed	Corolla 20–30 × 6–7 mm, red to orange-red, 10-lobed	Corolla 50–65 × 4–8 mm, deep blue to purple, 5-lobed
**Fruit**	Berry slightly ovoid ca. 1.2–1.4 × 0.9–1.1 cm, greenish-yellow when ripe	Berry markedly ovoid 1.2–1.7 × 1–1.5 cm, green and purple when ripe	Berry markedly ovoid, 1.3–1.8 x 0.9–1.3 cm green, reddish-brown or purple when ripe	Berry markedly ovoid, 1.6–2.5 x 1.2–1.7 cm, yellow, green or brown when ripe	Berry markedly ovoid to conical, 2.3–3.5 x 1.4–2 cm, white when ripe

The rediscovery of *I.orozcoae* over two centuries since the first collection by José Celestino Mutis in 1783 underscores the immense importance of historical collections in documenting and describing botanical diversity and their potential contribution in setting conservation priorities within biodiverse regions. This is a widely-debated topic that is gaining increasing attention over time (Besnard et al. 2018; Albani Rocchetti et al. 2021; Vargas et al. 2023). In the case of *I.orozcoae*, the presence of the long undetermined specimens spurred renewed collecting efforts in the eastern Andean cordillera of Colombia, which continue to be the source for many newly-described taxa, some of which have remained elusive for many years (e.g. Granados-Tochoy et al. (2007)). Given the challenges associated with locating and collecting narrow endemics such as *I.orozcoae*, it will often be necessary to base descriptions on scant material so that perhaps in future, the timespan between additional collections of new species does not span centuries, an important consideration in the context of the ongoing efforts to conserve biodiversity.

#### Additional specimens examined.

Colombia. Cundinamarca: Ubate – Lenguazaque Highway, 2600 m elev., 16 September 1947, *O.L. Haught 6188* (COL, US); Lenguazaque, vía Ubate-Lenguazaque, antes del sector Las Balsas, en cercanías de los farallones de Lenguazaque 5°20'0.25"N, 73°43'23"W, 2600 m elev., 04 September 2021, *A. Orejuela 3407 with H. Mendoza, J. Castillo. V. Luna, C. Luna & M. Mora* (JBB, COL, HEAA); Colombia, sin. Loc., 01 January 1783, *J.C. Mutis 636* (COL).

### ﻿Key to the Colombian species of *Iochroma*

**Table:** 

1	Corolla 15–70 mm long, tubular, flaring at the mouth, red, orange-red, orange, deep blue or purple, calyx conspicuously accrescent in fruit, fruit a slightly to markedly ovoid to conical berry	**2**
–	Corolla 8–11 mm long, campanulate-infundibuliform, white, greenish or greenish-cream coloured, calyx non-accrescent or accrescent to an insignificant degree in fruit, fruit a globose berry	***Iochromaarborescens***
2	Corolla red, orange-red or orange-coloured	**3**
–	Corolla deep blue or purple-coloured	**4**
3	Leaves usually pubescent; inflorescences with 30 to 120 flowers; calyx 3–7 mm long, cup-shaped, densely pubescent; corolla pubescent; anthers usually included	***Iochromagesnerioides***
–	Leaves usually glabrescent; inflorescences with 1 to 15 flowers; calyx 7–13 mm long, tubular to campanulate, glabrous or with a few scattered hairs; corolla glabrescent; anthers usually exserted or partially exserted	***Iochromafuchsioides***
4	Leaves (2.1–) 5.3–11.6 × (1.2–) 2.2–5.5 cm. Inflorescences few per branch, located near the shoot apex; flowers 1 to 4 (–8) per inflorescence; corolla 1.5–2 cm long, 5-lobed; berry greenish-yellow when ripe	***Iochromaorozcoae***
–	Leaves (6–) 9–17 × (2.5–) 3–6 cm. Inflorescences many per branch, typically in massive clusters on older, often leafless branches, rarely near the shoot apex; flowers 6 to 12 per inflorescences; corolla 2.8–4.5 cm, 10-lobed; berry green and purple when ripe	***Iochromabaumii***

## Supplementary Material

XML Treatment for Iochroma orozcoae

## ﻿Additional information

### Author ORCIDs

Andrés Orejuela https://orcid.org/0000-0002-3511-1478

Stacey D. Smith https://orcid.org/0000-0003-2010-4459

Boris Villanueva https://orcid.org/0000-0002-6929-3572

Rocío Deanna https://orcid.org/0000-0001-8753-7596

### Data availability

All of the data that support the findings of this study are available in the main text.
